# The mythconception of the mad genius

**DOI:** 10.3389/fpsyg.2014.00079

**Published:** 2014-02-26

**Authors:** Arne Dietrich

**Affiliations:** ^1^Department of Psychology, American University of BeirutBeirut, Lebanon

**Keywords:** availability heuristic, base rate fallacy, bipolar disorder, cognitive bias, creativity, illusionary correlations, madness, mental illness

Take troubled Vincent van Gogh, famed 19th century painter who suffered from bipolar disorder, cut off part of his left ear, and eventually committed suicide. Or Isaac Newton, eccentric 17th century physicist, general headcase, and judging from his leviathan superego, a candidate for making the diagnostic criteria of at least half a dozen psychological disorders. No sooner do we contemplate this aberrant pair, a whole army of mad geniuses springs to mind led by such illustrious figures as autistic Wolfgang Amadeus, depressed Ludwig van, or tortured Edgar Allan. Like Franz Kafka, Robert Schumann, Michelangelo, Virginia Wolf, Richard Strauss, John Nash, or Ernest Hemingway, they were all, at some point in their lives, anguished, tormented, alcoholic, angst-ridden, manic, outright psychotic, or just plain weird. Add the mind-boggling savant syndrome, throw in a quote from a venerable ancient Greek for good measure—say, Aristotle: “No great mind has ever existed without a touch of madness”—and we have the making of mythconception (for more details, see Rothenberg, 1990; Schlesinger, 2009, 2012; Simonton, in press).

Writers must by now have spilled gallons of ink over the purported link between creativity and madness filling shelves of books and articles (e.g., Post, 1994; Kaufman, 2005; Koh, 2006). Such tales from the insanity zone are nuggets of pure gold for the true believer in the unlock-your-infinite-creative-potential movement. What if we could just open “the doors of perception?” What would we have lost had Prozac turned Nietzsche into a regular bloke? Sadly, there is no sign that this kettle is going off the boil anytime soon. Hollywood can't get enough of it. Nor does the TED Conference, the new home of international meme laundering. The narrative of the troubled genius just strikes all the right chords for coverage in the tweet-sized attention span of modern news reporting. Not even the BBC can resist, having featured a headline last year reading: “Creativity ‘closely entwined with mental illness”’ (Kyaga et al., 2011; Roberts, 2012). Such frenzied enthusiasm and a few flag-waving generalization might be forgiven among those untouched by the purifying powers of statistical reasoning, but one would expect more professional scrutiny in the rarefied air of peer-reviewed psychology journals. Far from it. Even in the academic ether do respectable people, even those of the highest scientific standing, regularly rise to levels of speculation that can safely be called imprudent (see, for instance, Jamison, 1993).

So what, then, is the link? Is there indeed just a thin line separating insanity from genius? The best place to start looking for an answer, one would think, is the scientific literature—if that is the right phrase to use here. I say this because one quickly discovers, while perusing this literature, that there does not seem to be any scientific data on the matter. The entire thesis of the highly-gifted mentally ill rests entirely on an unholy marriage of case reports and anecdotal storytelling (see for instance, Jamison, 1993 or Post, 1994). It is not uncommon, for instance, to read articles galloping through so many esoteric live episodes, irrelevant factoids, and so much delicious gossip (did you know that the reclusive William Cavendish insisted on having a chicken roasting at all hours of the day?), that the validity of the link is all but a foregone conclusion. But it is one thing to be enchanted by folklore, it is quite another to turn a blind eye to lethal doses of selective data reporting.

Like no other field of psychology, the study of creativity is beset with nebulous concepts, combustible propositions and myopic theorizing, to say nothing of all the vacuous fluff out there. The fog enshrouding this particular Potemkin village is nevertheless easy to lift. We need only to drill into some basic numbers on mental illness that continue to be enthusiastically ignored—incidence and prevalence data, to be precise—take the wraps off an astonishing medley of cognitive biases—base rate fallacy, availability heuristic, illusionary correlations and the like—and unpack a few question-begging definitions of creativity.

As every undergraduate student knows, to establish a positive correlation between event A and some other event B, you need to collect baseline data on the frequency of both events. According to the (World Health Organization, 2013), mental illness, unlike genius, is by no means a rare phenomenon. Mood disorders, such as the various forms of bipolar and depressive disorders, occur in about 10% of the population. This amounts to hundreds of millions of people! Similar prevalence rates exist for anxiety disorders, which makes for a few more—wait for it—hundreds of millions of people! There is somewhat less suffering from schizophrenia, substance abuse disorder, the different kinds of personality disorders and autism, but enough to add several tens of millions more. The Centers for Disease Control and Prevention (2001) estimated in 2001—when the world's population stood at 6.2 billion, or about a billion less than today—that there were some 450,000,000 people living in poor mental health. The lifetime incidence of people making at least a single visit to the mind's Pre-Hell is said to be significantly over 50%.

What do these staggering numbers mean? In the somber land of regression curves, they tell us that we can spare ourselves the trouble of determining the frequency of eminence in the population. For, irrespective of how we define creativity, let alone genius, this number must be less—vastly less. The simple truth of the matter is that the VAST majority of creative people are not mentally ill and, more importantly, the VAST majority of those suffering from psychopathology are not geniuses. Seen in this light, the claim that creativity and insanity somehow go together sounds more like densely ignorant nonsense, the stunted idea of someone who spent too many hours in a hot tub.

It isn‘t my goal here to make a case for the opposite claim, but, by all evidence, it is hard to escape that conclusion. By the looks of these numbers, I would wager good money that the link between mental illness and genius is negative. To be exact: extremely negative. This isn’t to say that there might be something to it, perhaps if the data is parsed differently (see Simonton, in press), but this link, unqualified as in the BBC headline above, is wrong—outright! This would seems to hold not only for psychopathology *tout court*, but also for each psychological disorder alone, as well as, to restrict things further to severe cases of a given disorder or to specific types of creativity (Waddell, 1998). That this fact has been almost universally overlooked, like one would a tic, is as crazy as it is amazing.

Most psychology undergraduate students, if they are reasonably attentive, would recognize the mad-genius howler as a textbook case of the base rate fallacy (Kahneman and Tversky, 1973). This common statistical sin, also known as base rate neglect, concerns the tendency to focus on specific information and ignore generic, baseline information, even when—and here is the rub—the latter is presented. Thus, people greatly underestimate the probability of a genius being totally sane and greatly overestimate the probability of an individual with mental illness being creative. The fact is that a very large proportion of creative people have no pathological symptoms (Simonton, 2005, in press). Incidentally, the same reference point neglect occurs for insanity and violence (Stuart, 2003). This link, too, is strongly negative, despite the perception we get from the media.

But it doesn't end there. This error in thinking is so extensive and the opportunities for flummoxing so abundant that this matter is sure to continue to generate more heat than light. It is a disarming reflection of our reluctance, or inability, to think statistically that we just can't seem to snap, crackle, pop out of it. What makes our intuition misfire by such a wide margin? Seeing the world through our own warped force field is standard operating procedure of course. Psychologists have long accepted the sobering fact that our mind comes with a whole stack of cognitive biases preloaded and preinstalled. Without getting too technical about it, the one doing most of the dopamine squirting here bears the inauspiciously label “availability heuristic.” It is a mental shortcut that estimates the likelihood or frequency of an event by the ease with which a specific instance of it comes to mind. So when you think about the creativity-madness link, the odd behaviors of Michael Jackson are more likely to guide you than the 99% media-invisible normals.

The availability heuristic as a cognitive mechanism was first proposed and demonstrated by Kahneman and Tversky (1973). In a now classic experiment, they asked people to judge the likelihoods of an English word either starting with the letter K or having a K as its third letter. With people more readily thinking of kitchen, kennel or kickboxing than ankle, Eskimo or acknowledge, their participants overestimated the number of words starting with a K and underestimated those with a K in third position. An English text, however, has about three times as many words with a third-place K; they are just not as available in memory.

What's more, the availability heuristic also causes illusionary correlations, for the same reason. This leads to the perception of a non-existent relationship between two events simply because they occurred together at some point in the past (Chapman, 1967). Alternatively, this false impression can also arise from the way people incorrectly integrate contingency information (Perales and Shanks, 2007). Naturally enough, the more vivid the pairing, the more people tend to enduringly conflate the events and overestimate the frequency of their co-occurrence, and thus their causal relationship. The loopy logic then comes full circle with the confirmation bias, the tendency people have of confirming their existing beliefs. Cases that substantiate the belief, and ambiguous information that can be tweaked that way, strengthens the imaginary connection, while cases that violate or disconfirm it are ignored. Consider this rather typical finding from Redelmeier and Tversky (1996), who asked arthritis patients to track the weather over 15 months and judge to what extent their condition was related to it. While the correlation was actually zero, virtually all were certain that their level of pain depended on the weather. We have here a knockdown one-two punch then. The availability heuristic serves as the seed for the illusionary correlation between madness and genius, and the confirmation bias supplies the fertilizer that nourishes it.

I could go on and on. In fact, I think I will. Pulling conceptual rabbits out of metaphysical thin air is routine business in creativity research. Open any source, academic or otherwise, and you will find the concept of creativity linked to, say, low arousal, defocused attention, right brains, unconscious processes, lateral thinking, or altered states of consciousness, to name but a few popular themes, when common sense alone tells you that their opposites are also sources of creative thinking (Dietrich, 2007). Consider, for instance, a study by Kyaga and colleagues (2011) that searched the database of Swedish registries for the insanely gifted, as it were. The real humdinger of the study was the operational definition of creativity. They found mental illness to be more common in people holding “creative occupations“—artists, writers, and scientists—compared to the evidently insipid army of accountants and auditors. Not only would this be news to engineers in Silicon Valley, but also the authors ask us to accept that writers and graphic designers are—by definitional fiat—creative. This is nuts. For the record, this study is the one that led to the BBC headline quoted earlier.

All of this would seem to suggest that some serious scientific work needs to be done on the matter. In addition to controlling for cognitive biases, measurement and analytic issues can also contribute to a false assessment of the creativity-madness link (Simonton, in press). Until such time, I take my inspiration form the humanistic perspective and prefer to think, just like Abraham Maslow and Carl Rogers did, that creativity is associated with mental health. Standing tall at the top of the hierarchy of needs, creative imagination and expression is the hallmark of a well-adjusted, self-actualizing, fully functioning person.
